# Iowa’s Integrated Data System for Decision-Making (I2D2):  a collaborative approach for responsible and relevant use of population data

**DOI:** 10.23889/ijpds.v9i5.2903

**Published:** 2024-09-18

**Authors:** Heather Rouse, W. Todd Abraham

**Affiliations:** 1 Iowa State University

Integrated data systems (IDS) bring together people, processes, and priorities in a disciplined approach to generate evidence about child and family needs and service system gaps. In response to pressure for more efficiently coordinated early childhood services, Iowa built an IDS as a collaborative effort between university researchers and government leaders. Iowa’s Integrated Data System for Decision-Making (I2D2) includes an engaged governance approach that has received national attention as a model for ensuring federal and state priorities direct the work, rigorous scientific standards are used, and community engaged users have access to the information they need when they need it. This presentation will describe I2D2’s governance and technology solutions and provide examples of cyclical, community-engaged projects that have informed program improvement. Multi-stage integration protocols ensure individual records from disparate systems in health, education, child welfare, and workforce are accurately matched within a process that attends to equity. Dissemination processes within communities of practitioners, executive leaders, and researchers ensure findings are relevant and used to drive strategic planning. Public facing dashboards and interactive applications display prioritized indicators and support improved data literacy and common language across partner groups. Examples of use will be discussed including the evolution of a statewide strategic planning process using integrated data indicators, evidence-based expansion of home visiting programming to meet the needs of underserved families, and support for sustainable childcare businesses through the availability of real-time vacancy, supply, and demand data and easy to access applications for families to find available care when they need it.

